# The Psychological Implications of Companion Robots: A Theoretical Framework and an Experimental Setup

**DOI:** 10.1007/s12369-021-00846-x

**Published:** 2022-01-28

**Authors:** Nicoletta Massa, Piercosma Bisconti, Daniele Nardi

**Affiliations:** 1 Department of Psychology, Sapienza University of Rome, Rome, Italy; 2 DIRPOLIS Institute, Sant’Anna School of Advanced Studies, Pisa, Italy; 3 Dipartimento di Ingegneria Informatica, Automatica e Gestionale, Sapienza University of Rome, Rome, Italy

**Keywords:** Psychology of human-robot interactions, Companion robots, Sexual robots, Philosophy of robotics, Immersivity, Sexual robotics, Human–robot interactions, Anthropomorphic robots

## Abstract

In this paper we present a theoretical framework to understand the underlying psychological mechanism involved in human-Companion Robot interactions. At first, we take the case of Sexual Robotics, where the psychological dynamics are more evident, to thereafter extend the discussion to Companion Robotics in general. First, we discuss the differences between a sex-toy and a Sexual Robots, concluding that the latter may establish a collusive and confirmative dynamics with the user. We claim that the collusiveness leads to two main consequences, such as the fixation on a specific and atypical type of sexual interaction, called paraphilic, and to the infantilization of the user, which we explain through the theoretical framework of “object-relation theory”. We argue that these dynamics may degrade to an infantile stage the relational abilities of users, extending this argument to Companion Robots in general. Then, we enquire if and how the relational dynamics enacted in HRI may shift to human relations: we discuss the analogy with virtual reality concluding that, under certain condition, a symbolic shift might happen. In the last part of this work, we propose an experimental setup to verify if a collusive and confirmative interaction with a Companion Robot can, over time, impact on the user’s ability to manage relational frustration.

## Introduction

Over the last 20 years, the research on Social Robots (SRs), namely machines specifically conceived and designed to produce a social interaction with users, has gained growing interest both in the academic field and in the public opinion. Within this group of robots, often anthropomorphic both in appearance and behaviour, we can further distinguish *Companion robots*, designed to establish a relationship and an emotional bonding with the user.

So far, SRs are often designed for caring tasks towards socially fragile subjects with little or no social interactions. In fact, most of the research on this topic is focalized on the use of companion robots with elders [1] and people with autism spectrum disorder [2, 3].

The use of social robotics in healthcare is indeed one of the most promising fields for the application of this technology, which embodies an extremely interdisciplinary approach.

Moreover, the global COVID-19 pandemic is speeding up the process of integrating Companion Robots into society, especially the assistive ones for elders [4]. The same is true for another type of Companion Robot (CRs), the Sexual Robots (SexRs), namely machines built with the specific task of simulating an anthropomorphic sexual interaction.

As evidence of this, the global pandemic appears to have significantly increased the demand for anthropomorphic sex dolls and robots. This was announced by the Sex Doll Genie sector company, which explained that it received 52% more orders than the previous year from single men, while the requests made by couples in the same period increased by 33%.^1^

Certainly, Sex-Robots and sex dolls can also be used within sexually active couples, as reported by the company Sex Doll Genie, but the current academic literature continues to emphasize how this technology is mainly adopted by subjects with little sexual interactions with other humans. In this paper, we will discuss Sexual Robots’ implications as a paradigmatic example of the psychological consequences of Companion Robots in general, as we will show in the next chapters.

The academic discussion regarding the desirability of introducing Sexual Robots into the public market, has been rather intense so far. On the one hand, we can identify supporters of extremely polarized positions: among them certainly Katlheen Richardson, who started a campaign to ban Sexual Robots from the market [5]. On the other hand, other scholars maintain more prudent positions, considering the little experimental research on the possible effects of Companion Robotics done so far.

David Levy, the author of the ground-breaking book "Sex & Love with Robots" [6] was among the first scholars to draw the attention of the academic community to Sexual Robots. He, both in his book and in other papers [7], argues that the benefits of introducing Sexual Robots into society are certainly greater than the social, ethical and psychological issues raised by other scholars. In any case, a major concern of the current literature is that SRs can modify the social values and behavioural patterns of users, leading for example to socially degrade the vision of women [8], to increase the loneliness of users [9] and sexually violent behaviour [10].

However, to argue that Sexual Robots can change social values and user behaviour, a theoretical background is needed to justify the symbolic shift of social values produced by the human–robot sexual relationship in human society, such as the aforementioned increase in sexist behaviours; on the other hand, it is also necessary to theoretically justify the shift of relational patterns from human-Sexual Robot to human–human relationships.

The claim that a user who rapes his sexual robot will be more inclined, after a certain period, to increase violent sexual approaches even in sexual interactions with other human beings, is less obvious than it seems at first sight. John Danaher, in one chapter of the book “Robot Sex: Social and Ethical Implications [11], underlines some theoretical difficulties that the "symbolic-consequences approach" does not seem to overcome easily. In another paper, one of the authors of this manuscript already examined the various conceptual flaws of the current literature on the symbolic approach, as for example the work of Danaher [11] and proposed a first conceptual framework explaining the symbolic shift process [12]. In this work, that framework is deepened, improved and specified within a psychological approach that is based on the concept of “immersion” to explain the mechanism of transfer from HR to HH. Subsequently, the implications for the subject's organization will be outlined from both a philosophical and a psychological point of view.

We argue that the association between sex-toys and Sexual Robots is untenable, since the latter, through simulated relationality, leads to a symbolic immersion from which the subject derives the experience of gratification. We will also explain how this happens, emphasizing how the responsiveness of the artifact puts in place passive-confirmatory relational dynamics, collusive with the symbolic content of the subject. These elements are not involved in the use of sex toys and represent the indispensable requirements in structuring the quasi-other nature of the sexual robot.

We will also discuss the development of attachment and its bidirectional relationship with the affectivity and sexuality systems in an adult subject; subsequently, we introduce the "object relation theory". Through this theoretical framework, we argue how the interaction with a sexual robot and his collusiveness, can lead to a regression to infantile relational dynamics, therefore to a possible decrease in the ability to manage relational frustration. Lastly, to a potential paraphilic fixation in subjects in which sexuality is already atypical.

In the second chapter, we analyse the "Symbolic consequences argument"; we highlight the need for a theoretical framework that justifies the symbolic transfer to society and human–human interaction. To fill this gap, we propose a parallelism with virtual and augmented reality, inserting it into the theoretical perspective of embodied cognition. By paying attention to the role of immersion in recalling, reinforcing, or modifying specific behavioural patterns, we clarify how the simulation of the relationship, which can be experienced through the robot, can be similar to these other forms of simulation. This potentially explains the symbolic shift.

We discuss how our conclusion on “collusion” of simulated relations can be extended to relational artefacts in general, namely Companion Robots. In fact, Sexual Robots account for a clear example of the relational dynamics between relational artefacts and users. Yet the collusivity, and the other conclusions of this manuscript on relational frustration management, may be extended to Companion Robotics in general. Specifically, we will show how our claims are generalizable and how Sexual Robots can be regarded as a clear, paradigmatic example.

In light of this, an experimental setting will be proposed concerning what we hypothesized: namely whether an immersive collusive interaction with a relational artifact can lead the user to manage frustrating relational episodes less effectively. To do this, we decided to use the humanoid robot NAO and biofeedback, a psychophysiological intervention method used in this case to detect the psychological and somatic activation of the subject in response to stressful stimuli.

The main contribution of this manuscript is to offer a clear theoretical framework to understand how and why HRI may impact on human–human relations (HHR). Moreover, we design an experimental setup to verify our hypothesis.

Therefore, first is necessary to analyse the differences between common sex toys and anthropomorphic sex robots, starting from the wrong typical equation between them that is shared by most of the literature. In fact, from that point of view, the robot is seen as a simple object. Moreover, following this equation, there is not any possibility for the user of being deceived about the artificial nature of current robots, therefore human-SexR interactions consequences are not different from ones that bring a common sex toy.

Against this view, we will show how the relationship with Sexual Robots produces peculiar dynamics compared to both the interaction with Sex-Toys and with other human beings.

## More than a Sex Toy: Absence of Partialization and Simulated Otherness

In the collective imagination, sex toys, a category of objects built ad hoc to satisfy the erotic-sexual pleasure of an individual and/or any relational configuration, are today considered in their exquisitely playful dimension and find diffusion in a wide and variegated segment of the population.

Today their most ancient logic of use has been recalled, going beyond the previous and gloomy nineteenth-century vision that saw them as therapeutic tools meant for what were considered typically feminine disorders [13]. Over time, it became clear that what was erroneously labelled as "hysterical symptomatology" actually was the research of a healthier and more complete expression of one's identity in general and sexuality in particular, until then considered taboos to be repressed and a form of emancipation to be confined.

On the contrary, today their main focus is the exploration, play, stimulation and rediscovery of one's own sexuality [14]. From this point of view, sexuality is now no longer annihilated, but expressed: a relevant dimension contributing to the overall well-being of the person.

By this point and through a game of associations, the equivalent goals are considered to be traceable in another type of artifact. An artifact considered, in the aforementioned perspective, an advanced form of sex toy through which expressing one's sexuality and experiment new interpersonal dynamics: the sexual robot.

This perspective is well represented by the text by David Levy [6]. In this text, behind a factitious openness to experimentation and growth through the sexual dimension, there is a background where sexuality could be greatly simplified.

As we show, the absence of partialization and the presence of a simulated otherness, represent the main reasons why a synonymy between these artifacts is unthinkable in our opinion, especially since there is a peculiar dimension solicited by today’s sexual robots: the simulation of the relationship.

However, what makes the sexual robot different from any other artifact is not only the human characteristics and the responsiveness^2^ characterizing it, which also have a central role as we will see afterward.

Lack of partialization, that is the partial representation of genitality, would seem to include the sexual robot into the category of real dolls. It introduces a collusive otherness and an amplification of the erotic-affective imaginary of the subject into the simulated relational dimension, where it is now placed in.

Pleasure relies on the creation of a simulated complicity. Complicity that finds further reinforcement in the users’ freedom of choice between a set of different robot’s personalities, to make it as isomorphic as possible to their imagination.

Sexuality, far from being merely conceived as a set of drives to be mechanically realized, rather represents a space in which gratification involves relational dynamics, where the subjects participate in its co-construction. On the other hand, in this case the simulated relationality is characterized by a counterpart that interacts with a constitutively confirmatory setting towards the user. This results in a self-referential process in which the only “otherness” involved is the one reflected in the artifact, namely the user’s one.

This allows us to introduce a dual theme which, to some extent, would be in clear contradiction with the aim of those who identify SexR as a potential tool for amplifying possible erotic-affective experiences. Assuming that a part of the population interested in buying a SexR has an already atypically connoted sexuality [5], it is possible that the robot itself can become the object of a paraphilic attraction, which has precisely in the predilection of the atypicality of the sexual object, in this case anthropomorphized, its distinctive feature. But not only that: it could equally represent a landing and sedimentation point in interpersonal dynamics, far from the generativity characterizing the relation with another individual.

As we have seen, this interactional peculiarity of Sexual Robots is given by their peculiar relational posture within the relationship with the user: an element that leads the robot to configure a type of relationship at the limit between objective and intersubjective. On the one hand, the responsive and interactive nature of the robot would make the interaction similar to human–human ones. The fact that users, when engaged in an interaction with a Social Robot, adopt behaviours extremely similar to those expressed with other humans, is widely demonstrated by the literature on interaction studies [15–19].

This "anthropomorphization" of the robot at an interactional level increases together with the robot's ability to respond to visual, tactile and verbal stimuli. The ability to manage facial expressions following the verbal register; the ability to manage proxemics [17, 20] and, in general, the so-called non-verbal cues [21–23] are also essential issues. On the other hand, however, the robot ultimately remains an object available to the user.

Accordingly, we agree with Mark Coeckelbergh’s [24] and Sherry Turkle’s [25] reflections on the complexity of conceptualizing the nature of the relationship produced by the Social Robot. Following the words of Don Ihde [26], Coeckelbergh discusses how the Social Robot is, for the user, what can be called a quasi-other. The otherness of the robot, following Coeckelbergh's hypothesis, is finally a linguistic-symbolic construction that the user builds on the robot, linguistically exemplified in attributing to the robot the pronoun “s/he”, instead of “it”. In this sense, we must not consider the ontology of the robot (as an object or subject) important to establish the simulated intersubjective relationship, but the approach that the user has with the artifact. In the words of Coeckelbergh:

Interaction based on this appearance constitutes a (quasi-)social relation between us and the robot, regardless of the robot’s ontological status as defined by modern science and by traditional and modern metaphysics, which view the robot as a mere thing or machine.

Consequently, if the simulated interaction sets the stage for a somewhat intersubjective relationship between the user and the machine, this explains the "subjective" excess of the robot over the simple object. A step following this reflection makes the notion of quasi-other problematic on another side: if the robot remains a quasi-other, in which aspects does it not reach the stage of complete otherness? What crucial features of subjectivity are missing in the robot involved in an interaction with a human user? To discuss this point, we take for example users’ reactions interacting with relational artifacts in Sherry Turkle's experiment [25]: the comments of the little girl Orelia on MyRealDoll underline that the fundamental missing aspect experienced by the user is that the robot "is programmed to feel love". On the other hand, the elderly Andy interacts with the artifact as if it were her ex-wife. Orelia's comment indicates exactly the relational dynamics enacted by the artifact, and in our case even more strongly by the Sexual Robots, compared to the expectations of the user, for example Andy, in the interaction. What is established is a confirmatory dynamic of the user's relational expectations. What causes a form of distrust in Orelia may be what, in other subjects, can generate a strong attachment, namely the confirmatory dynamic. This may depend on the fact that the robot, like a relational mirror, reflects the user’s relational content and expectations, reinforced by the relational confirmation given by an interacting "other". In fact, the robot automatically colludes with the fantasies that the user builds on the human–robot relation, certainly returning a deformed version of intersubjective relationships.

This reflection is necessarily linked with the so-called “deception objection”, one of the most discussed issues of Companion Robotics [1, 27]. The discussion, multifaceted and complex, is often based on the possibility that the Companion Robot can "deceive" users about either its robotic nature or the "spontaneity" of its emotional and affective responses. We believe that this problem is a false flag even in the case of assistive robotics for elders. To put it in Turkle's words [25]:

At the same time, this kind of play with the doll does not necessarily mean that Andy is not aware that he is playing with a doll. Andy emphasizes that he knows the doll is a toy and not “really” alive. He is able to relate to the doll as a sentient other but also recognize it as an object.

In theoretical terms, in the current state of refinement of Companion Robots (and probably still for a long time), their simulation abilities will not allow them to overcome the quasi-other stage. While they will maintain an “excessive objectivity”, because of their relational abilities and because of the user anthropomorphization, on the other hand they will still lack those aspects allowing the experience of a true "relational otherness". This lack, however, can produce confirmatory and collusive dynamics towards the user's relational fantasies. The "deception objection" therefore seems to be a problem widely disconnected from the objective implications of Companion Robots, including Sexual Robots: at most, we could speak of auto-deception. In this case, more than an ethical or moral issue, this raises a question about the impact on the user's psychology. For this reason, we believe it is useful to address the issue reflecting on Sexual Robotics, which we consider a field of application where the confirmatory dynamics of the human–robot relationship can clearly show their implications for the user’s psychic organization. Obviously, the discussion on deception has not only psychological implications, but also ethical and normative implications. Is not the scope of this paper to expand the discussion to these other fields but to point out that, under a psychological lens, the relevant discussion is about the concept and the implications of self-deception in the case of collusive and confirmatory relational design of the robot.

From this point of view, we hypothesize that a paraphilic framework can be strengthened or established, setting users on a single mode of expression of their sexual-affective dimension, which would consequently be deprived and impoverished. Moreover, if we add an experience of ego-dystonic feeling, that is based on clinically significant discomfort, a real paraphilic disorder could be configured, significantly affecting the overall well-being of the subject.

In the next paragraphs we outline how the aforementioned dynamics can occur, highlighting the role of attachment and the usefulness of the “object relation theory”.

### Affective Stagnation and Infantile Object Relations

Erickson spoke about “stagnation” in his theory, centred on the dialectic between opposites that characterized every psychosocial development [28]. As opposed to generativity, stagnation represented the stasis of individual evolution, coinciding in this case with a renunciation of affectivity. These aspects concur to lead the subject away from a desirable integration and affirmation of the self.

The imprinting derived from attachment, and the indissoluble bond that it structures with sexuality and future adult affectivity, finds its place in this game of opposites, which start in the oxymoronic dynamic of trust-mistrust during childhood. A vast literature investigated and argued a strong and reciprocal influence between attachment strategies and sexuality: in fact, it is now possible to predict the type of interpersonal dynamics most likely to be identified in an adult subject, as well as the processes of emotional regulation and satisfaction of needs, even if not primarily sexual, where the sexuality can be a metaphorical representative [29–32].

The dissimulation of anxiety, to anticipate a possible rejection, is one of the elements found in people who were having or were interested in having, a relationship with an anthropomorphized artifact [33].

The minimization of affects in interpersonal dynamics are functional in limiting their potential deleterious effects [34–37].

In this context discussion, the refuge from "relational anxiety" [38] in the interaction with the SexR places the subject in what Steiner called a "psychic retreat" [39]. Here it is possible a dissociative adaptation, to shield the person from feelings of fear and loss, being under the direct control of the subject himself.

In this psychological context SexRs could find a place, representing a hook for those individuals who interpret the relationship with others as frightening. In fact, the artifact could act as a transitional object, with the seductive advantage of never exposing to loss and abandonment [40]. Therefore, the subject would be never exposed to that symbolic "transition" which would open to uncertainty but to full affectivity at the same time.

The aforementioned allows us to hypothesize that people with poor social interactions can be the most affected by this technology [41].

Accordingly, the simulated relationship will never be exposed to the risk of redefinition, unless there is a strong perturbation requiring a restoration of the internal coherence system [42], or a possible reframe where the relationship itself is questioned.

This is what would normally happen in a relationship between adult partners, in which only the most accurate and flexible strategies will generate adaptive and functional behaviours, producing a healthy and free relationship. A kind of relationality which, in order to present these virtuous characteristics, should allow for an integration between the internal motivational systems of sexuality, care and attachment [29]. Without these systems the mutuality, required for the well-being of the partners, would not exist.

This cannot happen in the human-sexual robot relation, in which sexuality could be recruited to serve the needs of attachment more than for those of mere erotic exploration. Nonetheless, a real bond with the other would still be missing [43], present only in a hallucinatory form and to the extent it is confirmatory for the needs of attachment.

The confirmatory effect of SRs on the user, as we have already claimed, is produced because the robot is a territory of projections generated by the user and by their self-referential nature. This will produce the artifact’s collusive dynamics, regarding both affective and sexual dimensions. Therefore, in contrast with Viik [44] argument, we claim that these aspects inevitably overcome the typically human steps that, in Viik’s opinion, are necessary to fall in love and establish a romantic relationship. In fact, shaped inside the user’s projection, the SR is then a perfect, stagnant bridge between safety and pleasure.

The confirmatory dynamics enacted in the human–robot interaction are what we believe to be the core of the infantilization concern regarding Companion Robot users, an aspect widely debated in the Companion Robotics literature [1]. While it is often poorly justified from a theoretical point of view, it remains an important ethical concern, especially in assistive robotics for the elderly. In the current discussion, the problem of infantilization seems to concern the loss of user’s responsibility, agency and privacy [45]. On the one hand, these topics might have important implications in the discussion on the implications of Companion Robotics on human rights. On the other hand, we want to emphasize how the infantilization process, due to interaction with a Sexual Robot (but generally extendable to Companion Robots), could have an important impact on the user's psychological organization. In fact, we will discuss how the infantilization process can be substantiated in the reiteration of a symbolic relational content which, through the artifact, is repeatedly performed and consequently strengthened. To understand how the confirmatory dynamics can lead to an infantilization of the user, it is useful to recall some concepts of the “object relation theory”. Specifically, we apply Donald Winnicott's theoretical approach on the differentiation, in the infantile phase, between internal and external object and the consequent passage from "relating with identification" to a dynamic of "use" [46]. The use of the Winnicottian approach in this context is particularly useful as it describes the evolution from an object relation to intersubjective relationships.

In the text "The use of an object" [46] Winnicott describes the passage, particularly evident in children, from an infantile to an adult relational setting, until the development of functional intersubjective relationships. This step is conceptualized by Winnicott in the difference between "relating with identifications" and the "use" of an object. The first is characterized by the indistinction between the internal object (a projective and fantastic production), namely the representation that the subject makes of the object, and the external object, independent and therefore potentially cause of frustration [47]. At this stage, the experience of object relation only concerns the subject projections since the external object, in the psychic experience, remains in fact non-existent, a bundle of projections [48]. At this stage, if the object—whether it is an inert entity or a human being—produces frustration, namely it does not conform to the subject’s desires, the infant will attack the internal object which now no longer fits the fantasy. To visualize this dynamic, it is sufficient to think of children frustrated by the absence of the mother's breast, if they are hungry. If the caregiver restores a frustration-free stage, the child's aggressive strategy will have success and the autonomy of the external object with respect to the infant's imagination will be cancelled, the dominion of phantasy restored. On the other hand, the caregiver may resist the aggression, a positive characteristic of the "good-enough-mother". In this case, the aggressiveness of the infant will not have an effect on the external object, in this case the mother. The survival of the external object—namely the persistence of the frustrating situation, destroyed in the imagination—will produce a gap between phantasy and reality, a discrepancy between the internal and external object. This residuality will attest to the child the independence of the object from his own imagination, releasing for the first time the object from the domain of phantasy omnipotence. This supports the "use" of the object and the establishment of intersubjectivity, namely the understanding a respect of the autonomy of the other in respect to our phantasy. Winnicott concludes [46]:

This thing that there is in between relating and use is the subject's placing of the object outside the area of the subject's omnipotent control, that is, the subject's perception of the object as an external phenomenon, not as a projective entity, in fact recognition of it as an entity in its own right

On the same line of reasoning seems to be Liberati [49] when he claims:

The digital other in the everyday world calls for an intertwinement between its actions and the actions of the subject in the everyday world. This co-action in the everyday world is what makes the “other” resistant. Thus this “digital other”, even if it has a digital content, is perceived as “resistant” from the subject.

This “resistance” is in line, from a philosophical perspective, on what we claim through Winnicott about the importance of the construction of a difference between the internal and external objects. The recognition of the object residuality with respect to the internal representation, and therefore of its structural independence, is at the core of a functional intersubjective relationship also in Jessica Benjamin [50, 51]. Intersubjective relationships require a continuous exchange of the position of doer/done-to, which describes the dialectical relationship between recognition of the other and omnipotence fantasies.

“The recognition process occurs when the subject and the other […] are conceived as always mutable mirrors reflecting the interlocutor, so that this reflection is neither mimetic nor annihilating of the parts at play, but rather allows for a continuous and permanent interchange of polarity, not fixed in an oppositional (doer/done-to) form" [translation is mine] [52]

Finally, the transition to the stage of "use" of an object is not only the enabler of intersubjectivity, but also what allows a subject to tolerate relational frustration, namely the structural independence of the other from his own internal representation [53].

Starting from these theoretical coordinates, it is therefore necessary to reflect on the dynamics that may arise in human-SexRs interaction. First of all, we highlight that the confirmatory dynamics of the robot must not only be understood as an effect of the simple responsiveness of the relational artifact. Not every responsive interaction is a confirmatory interaction, but certainly no confirmatory dynamics can exist without a responsive interaction. What is "confirmed" in the H-R interaction is ultimately the adherence of the external-object-robot to the internal object of the user's imagination, obviously on different projective degrees depending on the user. The collusive structure of the relationship between Andy and his robot is not primarily due to the robot *actively* providing some specific interactional content (purring rather than saying "I love you"). On the contrary, it is because the robot cannot *avoid* colluding with the hallucinatory content of the interaction (e.g. the fact that the robot is Andy's ex-wife [25]), as it is unable to refuse it. In short, the robot adheres to the internal object since it is unable to manage the user's projective content on the relationship. We could, in a sense, call this process a "passive collusion with the relational fantasy of the user". At the current stage of technical advancement in relational robotics, this is the dynamic that most likely can occur in H-R interactions, even if first examples of active collusion begin to exist^3^ already. In conclusion, the robot’s passive collusion promotes a regression of the external object into indistinction with the internal one, on which the subject is omnipotent. This regressive aspect describes the user's "infantilization", which can therefore decrease the ability to tolerate relational frustration. This last aspect is in fact intimately connected with the ability to tolerate that the object does not conform to the user's imagination and interactional expectations. In our opinion, these are structural implications of Companion Robots: the actual design of Sexual Robots, aiming to have a market success, only amplifies the “passive collusion” of the artifact. In any case, as long as the robot is structurally a quasi-other in the relation, these consequences pertain to CRs in general. Also Bergen [54] enquires the implications on users of robots’ quasi-other design, in this case from the perspective of phenomenology of Eros of Levinas. He claims that until robots will not produce an alterity relation they will produce objectifying human-sexbot relations.

Therefore, the confirmative dynamic of the robot is in the first instance produced by the relational artifact responsiveness. As previously outlined, this aspect makes HRI different from object relations with the sex toy, shaping the robot, from the point of view of a user like Andy, as a quasi-other. The confirmative dynamic enacted by the robot, however, causes infantilization when we understand it as collusion—passive or active—with the user’s projective relational content. We believe that the regression to an infantile relational setting of the distinction between internal and external object may, in some users, decrease the ability to tolerate relational stress. This, as argued in the next paragraph, could lead to the user’s fixation on a collusive relational setting, devoid of the aspect of internal renegotiation necessary for intersubjectivity. This could crystallize a more distinctly paraphilic framework.

### Beyond the Transgression: on the Risk of a Paraphilic Fixation

We mentioned how different psychological and socio-cultural dimensions concur to the expression of human sexuality [55]. Moreover, human sexuality is conceptualized along a continuum [56] starting from sexual normativity/typical sexuality on the one hand, understood from a statistical and non-moral point of view. On the other hand we find the categories belonging to the atypical sexuality such as transgressive practices, paraphilias and the sex offender that represent the extreme point of the spectrum.

Within this path sexuality unfolds, passing through transgression, meant as sexual practices that lie outside of the mainstream and that can enrich people’s erotic dimension, and paraphilic attractions.

This latter in particular is intended as an erotic interest in unusual dynamics that presuppose an atypia of the meta-sexual object (fetishisms, paedophilia) or enjoyment through pain (algolagnia), up to a configuration that can be associated with a real paraphilic disorder. In this case the term “disorder” was added to DSM-5 [57] to indicate a paraphilic interest that is a case of distress or impairment to the individual or a paraphilia whereby satisfaction entailed personal harm, or risk of harm, to others as in the case of sexual offending.

Overcoming the Freudian vision of sexuality as a dimension predominantly constituted of instincts and discharges [58], other visions find in our conceptual framework a better location, allowing us to clarify the modality through which a certain relational schema finds confirmations in its reiteration [59], in its cyclical staging, in this case through sexual robots. These artefacts could become the perfect territory for the enacting of different types of objectual bonds, which, supposedly, are especially of the paraphilic type.

It is certainly true that atypical dynamics can also be put in place within a human–human relational configuration, but the risk of erotic-affective fixation in paraphilic interests does not occur in this case. The substantial difference lies in the mutual agreement between the individuals involved in a reciprocal relationship. This can happen in the previously mentioned co-construction, which mediates two or more individual experiences that are not necessarily complementary in their fantasies. In this mediation, a gap in the realization of an erotic experience is possible, as we discussed within the “object relation theory”. This gap between the two phantasies will require a reformulation, a new narration co-constructed under the lens of sharing. Finally, the enjoyment relies precisely on the need of a renegotiation, inside the relational configuration in which the subjects meet each other.

Furthermore, a transgressive experience differs from a paraphilic setting: while it embodies an unusual content—external to the social categories of normativity and which finds expression in sharing—it does not configure "an intense and recurrent excitement" [57] deriving from that specific erotic behaviour, which instead properly defines paraphilia.

Given the above and the previous remarks on the confirmatory posture that the SexR assumes in the relational configuration with the user, what is missing would be exactly that element that prevents the return to a regressive object-bonding in which the otherness of the other is totally available: the absence of a symbolic renegotiation of the relation, which instead finds confirmation in a self-referential circularity.

For this reason, in relating with the SexR, certain recursive patterns might be atrophied, making progressively more difficult to self-inhibit the aforementioned paraphilic behaviours. Therefore, the Sexual Robot can degrade the ability to exercise a higher capacity for self-monitoring, mediated by meta-cognitive processes [60, 61], namely “any knowledge or cognitive activity that takes as its object, or regulates, any aspect of any cognitive enterprise” [62].

In addition, important considerations come from more markedly cognitive-behavioural approaches [63], in which situational factors, therefore learning-related, and the neurophysiological factors underlying behavioural disinhibition, are considered equally important in understanding how an atypical sexual behaviour is structured and reinforced.

We claim that in this dynamic a paraphilic fixation can occur. As already mentioned, it could have as primary focus either the artifact itself or the specific erotic dynamic reiterated by the subject. This dynamic will be validated by the robot’s simulation of interaction and its passive collusion. Supported also by the discussion from the previous chapter, it is possible to hypothesize that a preference for this interactional setting may involve a risk of disengagement in the search for real interpersonal dynamics, as also highlighted by other scholars [9, 64]. Moreover, a reinforcement of the paraphilic relational setting may follow, with potential generalization even outside the human-SexR relation.

## Immersivity and Collusion at the Core of the Symbolic Shift

As we have preliminarily mentioned in the introduction, a substantial part of the academic discussion on Sexual Robotics focuses on underlining how their introduction in the human social context can lead to transformation in the human sociality. The main hypothesis is the transfer of behavioural patterns, peculiar to the human–robot interaction in human–human relationships and, ultimately, modify social values and symbols. In the chapter “The symbolic-consequences argument in the sex robot debate” [11], John Danaher summarizes various positions on the so-called symbolic-consequences argument, outlining its substantially invariant structure:

1. “Sex robots do/will symbolically represent ethically problematic sexual norms. (Symbolic Claim.)
2. If sex robots do/will symbolically represent ethically problematic sexual norms, then their development and/or use will have negative consequences. (Consequential Claim.)
3. Therefore, if the development and/or use of sex robots will have negative consequences, we should probably do something about this. (Warning Call Conclusion)”

As Danaher points out, the most problematic passage of this structure is the second, pretending a transfer of the problematic relational setting of human–robot sexual interaction in the human social and relational sphere.

Quickly retracing some approaches to the symbolic argument, that we discussed in detail elsewhere [12], the current theoretical frameworks do not seem able to give an effective explanation on the possibility of the symbolic transfer process. There are countless examples in which subjects find themselves in a fictional environment, such as a theatre performance, without transferring the behavioural mechanisms out of that context. While Peeters & Halsager [65] and Gutiu [8] seem to adhere to a strictly consequentialist view of the symbolic transfer process—if SRs reinforce a sexist symbolic content, this will also be transferred into society—Richardson, a major advocate of the symbolic consequences of the SRs, does not give a precise explanation of this process [5, 66, 67]. Therefore, in our opinion, there is still a strong epistemic uncertainty about the validity of the symbolic-consequences argument. We provide a possible framework in the next chapter proposing a comparison between Sexual Robotics and the immersive experiences allowed by Virtual Reality (VR) and Augmented Reality (AR). This theoretical framework makes it possible to understand under which conditions and for which reasons the transfer process can take place.

### Immersivity as a Requirement of Transferability: Virtual and Augmented Reality

Sense of presence and emotional engagement: these are the requisites on which the power of a simulation is strengthened.

Virtual reality (VR) can be regarded as an advanced form of human–computer interface, which allows to interact and immerse into an environment specifically generated and similar to a real context [68]. Augmented reality (AR), on the other hand, adds new information to reality. These technologies enable the process of illusion from which that transformative perception of "feeling really there" is derived, in that new and elaborate dimension [69].

This dimension is perfectly at the service of that metacognition process typical of the human mind, understood as that self-reflective property that makes it capable of thinking itself, and therefore the cognitive-emotional processes that characterize it. VR has been documented to provide a greater self-reflectiveness than the one that can be experienced through the evocation of a memory or through classic visualization techniques [69]. This is justifiable in the theoretical perspective of embodied cognition [70], because VR and AR insert a fundamental dimension, namely the ecological-contextual one in which the subject is actually immersed. Furthermore, the embodied cognition theory documents that cognition is the result of the experiences we make. Our body is endowed with sensory-motor skills: these interact with the wider environmental context and are modified by it, in turn soliciting a process of mutual co-construction. The simulation experienced in this way makes VR an "advanced imaginative system" and an "embodied technology" [71, 72]. It induces a change at the level of cognitive processes and in particular of the perception of space and of the body of the user [73].

The power of this tool, which makes it extremely innovative in a psychotherapeutic setting, resides in the lever that creates between the perception that the subject has of reality and his system of assumptions or expectations about it and which are, sometimes mistakenly, conceived as coincident [74].

This separation between the plan of happening and the plan of signifying what happened, can emerge precisely through that sense of presence that the subject experiences through the perception of the simulated context, if s/he is accompanied in doing so by a properly trained professional. In this sense, it will be both through the elective means of simulation and through the guidance of the therapist that it will be possible to question, in vivo, one's own system of internal coherence. This may lead to re-signification of the activating event with a different narrative, which provides a greater level of well-being [75, 76]. The VR experience will also solicit the ability to observe one's thoughts and emotions during their emergence, facilitating their monitoring and consequent regulation according to the context, relational and otherwise: characteristics of the aforementioned metacognitive capabilities.

It is this disconnection between the dimension of perception and the symbolic one, that we hypothesize will not occur interacting with sexual robots. Such interaction, as we argued, becomes the canvas of self-referring projections of the subject's imagination, thus keeping it in a self-reinforcing dynamic, the effects of which we argued above.

Immersivity, thus constructed in the likelihood of reality and for its ability to stimulate as many sensorimotor channels as possible, also soliciting individual metacognition, is therefore what lies at the basis of the process of transferability. This is the clinical effect sought by cognitive-behavioural approaches [77]. On the other hand, the immersivity allowed by the Sexual Robot results in the strengthening of the user’s atypical relational dynamics, which we claim become potentially transferable.

More specifically, transferability is caused by the support that virtual reality provides to the learning process which, through it, becomes experiential [78]. Therefore, the transfer possibilities that simulation guarantees—to create a virtuous cycle characterized by interaction, thought and active experimentation—is in fact the reason why VR is one of the possible therapeutic tools used both for the treatment of phobias [79] and for the training and the enhancement of specific skills to transfer outside the simulation [80].

VR is therefore used to facilitate learning in a clinical setting which occurs in an extremely specific environment: close to reality and above all controlled. In this context one can experience one's own emotional-cognitive processes usually experienced in that specific activating circumstance, as well as the more distinctly physiological processes that accompany the experience [81, 82]. This allows the user to recognize, inhibit and secondarily modify them. It therefore represents a safe ground for personal exploration, which at best leads to an improvement in the quality of life of the subject but, as we discussed, it is not a step that occurs autonomously but under the guide of experienced psychologists.

For these reasons—namely the characteristics of (1) immersivity and (2) transferability outlined—we hypothesize an equivalence with sexual robotics, which can therefore be considered a declination of "advanced imaginative systems", in which the simulation target is the relationship.

As for VR, there is a strong immersivity and therefore both a sense of presence and involvement on the part of the user who relates with the artifact. To this is added, however, a further implicit reinforcement generated by the passive collusion that the artifact itself inevitably has, due to its relational posture, previously discussed, in the dialectic relation with the subject.

In fact, lacking a real external hook that would facilitate a co-constructed redefinition of the user's projective content—such as a partner or the therapist—the simulation ends up constantly repeating and reinforcing itself.

Also in the case of VR and AR there might be a transfer of symbolic content outside the simulated reality. In fact, VR is used not only in a clinical setting but also for ludic purposes. As an example, the user may have the chance to use VR sex games, which have been related to an increase in aggression and sexist biases [83, 84]. Similarly to what we claim about SexRs, if the partner in VR reproduces the same collusive-confirmatory dynamic towards the user, we believe that a similar effects highlighted in our work can occur, albeit with reduced intensity.

We believe that this difference in intensity is due to a higher object mediation present in the VR experience. For example, transition from daily life to simulation is much clearer than in the case of VR, with respect to SexRs: in VR experiences it is necessary to start the game, wear a headset, use a controller etc. Moreover, the implementation of physical stimuli is currently under development.

Moreover, the embodiment of the robot amplifies the characteristics of immersion and transferability of virtual reality. In fact, the artifact shares the experiential context with the user without presupposing an objectual mediation. These two differences, namely lack of objectual mediation and embodiment of the robot, will supposedly increase the process of immersivity and transferability outside the simulated relation. The concept of “mediation” has been thoroughly investigated in another paper [12] by one of the authors of this manuscript, to describe how the presence of mediation (objectual, contextual, normative) is able to impede the shift from simulated relation to human–human relations. On the other hand, the embodiment of the robot can be understood as a feature that reduces the level of mediation in HRI in respect to VR. The role of embodiment in HRI is of fundamental importance for empathetic interaction by the user toward robots [85]. Moreover, the role of embodiment is a key aspect also in the perceived social presence of the robot [86] and on the item of “trust” [87].

Therefore, when objectual mediation is reduced, as in the case of embodied robots, we expect higher levels of immersivity causing a higher chance of transferability.

In conclusion, the reiteration of certain symbolic contents, projected on the sexual robot and passively validated by it, facilitates that immersive process in which the relationship, sexualized or not, acts as a bridge for the symbolic shift that we suppose occurs. This shift, as above argued, involves a paraphilic fixation and a regressive effect on user’s relationality.

## A Possible Experimental Setting

In this last section we present and discuss a possible experimental setting that can validate the symbolic-consequences claim, as reported by Danaher, for the specific case of the ability to tolerate relational frustration in companion robots’ users. Here, we provided a theoretical background that addresses the question “can human–machine interactions have implications for human–human relationships?”. As above argued, Sexual Robots are only a paradigmatic case of this issue, that applies in general to Companion Robotics. Therefore, this experiment will involve a non-sexual relational artifact, aiming to generalize to CRs our conclusions on collusion. In this last part of the manuscript, we discuss what is, in our opinion, a possible way to validate the theoretical framework we have presented.

### Research Questions and Hypothesis

One of the elements we discussed extensively is the collusion of the robot with the user's relational fantasies. It is this collusion that we primarily connected to the possible "regressive" implications of the interaction with the Companion Robots (and Sexual Robots). The consequence of this collusiveness, in the long term, could be a decrease in the ability to sustain relational frustration, or to tolerate frustrating situations for the subject during an interaction, as previously stated. The hypothesis to explore is therefore that an immersive collusive interaction with a relational artifact can lead the user to manage frustrating relational episodes less effectively. Considering the substantial impossibility of putting into practice an experiment with Sexual Robots, partly due to the difficulty in finding such artifacts and partly due to the ethical implications that such an experiment could have, we propose to use Companion Robots. In fact, as we have already discussed, regressive dynamics from an interpersonal point of view generally concern any interactive artifact that (a) puts into practice a collusive dynamic with the user (b) produces a certain degree of relational immersivity. Hence, the aim of the experiment will be to verify whether a collusive interaction by a Companion Robot has effects on the user's ability to tolerate relational frustration in a human–human interaction.

### Experimental Setup

To validate our claim, we therefore propose to put the following experiment into practice: a number of participants are asked to use a NAO Personal Trainer for two weeks, to encourage physical exercise. Each participant is asked to train under the guidance of the NAO every day for 14 days. At the beginning and at the end of the two weeks participants will meet with a human personal trainer to evaluate their athletic abilities. The interaction with the personal trainer will be deliberately frustrating for the participant (providing negative feedback on the exercise performed by the user) both at the beginning and at the end of the two weeks. The degree of activation of the subjects will be measured using biofeedback, a non-invasive technique, used both for therapeutic aims and diagnostic scopes, that uses sensors attached to the body for measuring key body functions such as blood flow, blood pressure, heart rate and muscle tension that are strongly affected by emotional activation and stressful circumstances.

First, it will be used one of the assessment tools in biofeedback called psychophysiological stress profile which is often used in order to identify the reactions of all different bodily functions on physical, emotional or cognitive stressors. The evaluation of the stress profile provides indicative representation of the physiological parameters that are out of balance and more sensitive to stressors.

Lastly, the psychophysiological stress profile will be evaluated at the end of the experiment as it follows.

During the 2 weeks the participants will be divided into two groups. In the first group, the NAO will provide an extremely positive and collusive interaction, with constantly positive feedback with respect to training and, in general, to the user. The control group, on the other hand, will be provided with a NAO with a neutral attitude, which does not produce any positive reinforcement towards the user neither from the point of view of feedback on training nor in general as a social interaction. In the second meeting with the personal trainer who, again will produce a frustrating and negative interaction, the degree of activation will be measured again through biofeedback.

At this point we expect to see a major reactivity in the psychophysiological parameters that were measured at the beginning of the training, but only for the people who interacted with the positive NAO.

If the hypothesis guiding this experiment is correct, the group that interacted with the collusive NAO will have a significant increase in the degree of activation, following the frustrating human–human interaction, when training with the collusive NAO. On the other hand, we expect no particular changes on the control group between the first encounter and the second one. What we expect to happen is not a change, in just 2 weeks, of the subject's frustration management structure. On the other hand, it is likely that within the specific experiential field of feedback regarding physical training, this difference may be visible. Nevertheless, structural and massive effects could be observed only in the case of longer periods of collusive interaction with the robot, probably with more advanced machines than those currently available. We plan to run the experiment in the next future, with the aim of validating the theoretical framework developed in this paper. Moreover, the experimental setting needed a theoretical framework that correctly justifies our hypothesis, allowing us to interpret the eventual results. This theoretical framework, as we showed, was absent and this contribution’s aim primarily was to fill this theoretical gap.

### Conclusions: Relational Simulators

Throughout the paper, we proposed a reinterpretation and in-depth analysis of the "symbolic-consequences approach" starting from a preliminary differentiation between Sexual Robotics and the category of Sex Toys, to which it is normally associated. We first highlighted how the responsiveness of the artifact, and its inevitably collusive posture with respect to the user's imagination, help to facilitate a process of self-deception. On this process we believe both the simulation of the relationship and the potential self-reinforcing circuit of the regressive or paraphilic interpersonal dynamics are enforced and strengthened.

Regarding the possible regressive interactional dynamics, we have proposed an excursus that examines the reciprocal influences existing between the systems of attachment and sexuality. We underlined how SexRs act, in this perspective, as transitional objects, without ever exposing to relational frustration and loss.

Within this "psychic retreat" we believe it is possible the occurrence of a regression to an infantile stage, with a lack in distinguishing between internal and external object. This may divert the user from a functional relational setting, supposed to mediate individuals who participate in a relation by co-constructing it.

The absence of a “symbolic renegotiation” within the interpersonal dynamics also allowed us to explore the theme of atypical sexuality, and how a reinforcement or the emergence of a paraphilic picture can occur in users.

To support this hypothesis, cognitive-behavioural theories have been mentioned, which specified how the recursive play out of a certain pattern, in this case paraphilic, can lead to its atrophy, making it more difficult to inhibit through the involvement of meta-cognitive processes.

Therefore, assuming that in some circumstances the above situation could occur, we introduced the theoretical framework of embodied cognition. This framework explains how this can actually occur and might be then generalized also in human–human interactions.

Then, we compared the results obtained from the analysis of the literature regarding VR and AR and hypothesized a similarity between these "advanced imaginative systems" and sexual robotics.

We consider sexual robotics an advanced imaginative system and an embodied technology like virtual reality, creating an immersive scenario in which the simulation is governed by the threads of individual self-deception. We supposed that the simulated relationship—isomorphic to the symbolic level of the subject—can become for the user a potential mean of relational regression, or a tool to reinforce paraphilic sexual dynamics.

We believe that our conclusions can be extended, in their theoretical framework, to human-Companion Robot interactions in general, of which Sexual robots are a significant example. Interactions with a Companion Robot could lead to the same consequences of interpersonal regression, for example regarding Companion Robots caring for the elderly. In fact, if they produce a collusive interaction as reported by Turkle [25], they would produce the same implications by decreasing the user's ability to tolerate a subsequent frustrating situation from a relational point of view.

Obviously, the level of impact of robot’s collusiveness on human ability to tolerate frustration is deeply influenced by the percentage of HR collusive interactions in respect to non-collusive interactions. Since some social groups, as elders in care facilities or the so called “hikikomori”, have scarce social interactions in their case there is a high risk that HR collusive interactions might produce a modification of user’s relational abilities.

This theoretical hypothesis could be examined through an experimental design, that we presented in the previous paragraph.

Finally, a last and brief mention should be made of the potential therapeutic use of these artifacts within a clinical setting.

Currently, only data from interviews conducted with therapists and doctors are available in the literature. These studies enquire the possibility of using sexual robots for the treatment of specific diseases, especially in case of a strong symptomatic component associated with anxiety [88].

Therefore, as today there is no concrete confirmation of these clinical hypotheses, the research of the next few years should follow this direction. Currently, no conclusions can be drawn in the absence of experimental data, deriving from solid clinical settings.

There are some limitations in this work that we would like to point out: first, it deals only with an androcentric perspective on the issue of Sexual Robotics. This is due to the massive presence of gynoid Sexual Robots, while the android versions are far less developed also because of the perceived market demand [89]. The analysis of the relational setting between a sexual android and a woman could probably produce a partial reframe of the argument of this paper, while the conclusions could supposedly remain the same.

The second limitation of this paper is that it does not deeply analyse the possibility that Sexual Robots will be fetishized for the very fact that they are not humans. This would bring to a strong reformulation of the symbolic argument and its consequences for this paper, since a basic assumption at the ground of the symbolic approach is that users want to interact with Sexual Robots because they resemble humans. However, it is hardly possible that a large part of SRs users will be sexually attracted by the very fact that the robot is a machine and not a human: in this case they would choose to fetishize a non human-resembling object. In any case, the conclusions of this paper will be limited only to those users who use Sexual Robots as a symbolic substitute of a HHI sexual interaction.

## Footnotes

1 https://www.forbes.com/sites/frankicookney/2020/05/21/sex-doll-sales-surge-in-quarantine-but-its-not-just-about-loneliness.

2 [6] https://www.huffpost.com/entry/roxxxy-robot-girlfriend-l_n_418797 (accessed 03/12/2021).

3 http://mastersofmedia.hum.uva.nl/blog/2017/10/25/being-friends-with-yourself-how-friendship-is-programmed-within-the-ai-based-socialbot-replika (accessed 03/10/2021).
